# Evidence for a second regulatory binding site on PspF that is occupied by the C-terminal domain of PspA

**DOI:** 10.1371/journal.pone.0198564

**Published:** 2018-06-15

**Authors:** Eyleen Sabine Heidrich, Thomas Brüser

**Affiliations:** From the Institute of Microbiology, Leibniz Universität Hannover, Hannover, Germany; Centre National de la Recherche Scientifique, Aix-Marseille Université, FRANCE

## Abstract

PspA is a key component of the bacterial Psp membrane-stress response system. The biochemical and functional characterization of PspA is impeded by its oligomerization and aggregation properties. It was recently possible to solve the coiled coil structure of a completely soluble PspA fragment, PspA(1–144), that associates with the σ^54^ enhancer binding protein PspF at its W56-loop and thereby down-regulates the Psp response. We now found that the C-terminal part of PspA, PspA(145–222), also interacts with PspF and inhibits its activity in the absence of full-length PspA. Surprisingly, PspA(145–222) effects changed completely in the presence of full-length PspA, as promoter activity was triggered instead of being inhibited under this condition. PspA(145–222) thus interfered with the inhibitory effect of full-length PspA on PspF, most likely by interacting with full-length PspA that remained bound to PspF. In support of this view, a comprehensive bacterial-2-hybrid screen as well as co-purification analyses indicated a self-interaction of PspA(145–222) and an interaction with full-length PspA. This is the first direct demonstration of PspA/PspA and PspA/PspF interactions *in vivo* that are mediated by the C-terminus of PspA. The data indicate that regulatory binding sites on PspF do not only exist for the N-terminal coiled coil domain but also for the C-terminal domain of PspA. The inhibition of PspF by PspA-(145–222) was reduced upon membrane stress, whereas the inhibition of PspF by PspA(1–144) did not respond to membrane stress. We therefore propose that the C-terminal domain of PspA is crucial for the regulation of PspF in response to Psp system stimuli.

## Introduction

Many proteobacteria possess the phage shock protein (Psp) system that is upregulated under various stress conditions that can harm the cytoplasmic membrane [1,2]. In *Escherichia coli* and other enterobacteria, the Psp components are encoded by the *pspABCDE* operon and the monocistronic *pspG* gene. The expression of these genes depends on σ^54^ that is regulated by the PspF component, an enhancer binding protein divergently encoded upstream of the *pspABCDE* operon [3,4]. PspF in turn is regulated by PspA, the first product of the *pspABCDE* operon [5,6]. It is believed that PspA is not only a key regulator but also a membrane-stabilizing effector of the system [2,7]. Also PspB and PspC, two membrane proteins encoded within the *pspABCDE* operon, play an important role in signaling [8], and just like PspA, also PspB and PspC can have effector functions [9,10]. It is still unknown how exactly a stress signal is sensed and transmitted to PspF. Clear is that PspB and PspC interact in the membrane, and that the C-terminus of PspC in turn interacts with PspA upon a stress signal and thereby recruits PspA to the membrane [8]. The current opinion is that this membrane-recruitment leads to a dissociation of PspA from PspF, which activates PspF and thereby induces the Psp response [11]. The membrane interaction of an N-terminal amphipathic helix (AH1) of PspA is reported to be involved in stress sensing [12]. In the reported X-ray structure, this helix folds back to a coiled-coiled domain, which likely represents the resting state [13]. The interaction of this helix with membrane surfaces in response to membrane stored curvature elastic stress could be a membrane stress sensing mechanism [14,12]. Clearly, PspA is one of the key players in the regulation of the Psp response. Due to the auto-regulatory circuit, experimental approaches to study the native Psp regulatory cascade have limitations *in vivo*. Experimental work on PspA is further challenged by its tendency to aggregate and by the complexity of its numerous interactions. Fragmentation approaches proved useful to overcome some of these problems. Such approaches identified (i) determinants for PspA oligomerization in the C-terminal domain [15,5], (ii) the PspF-regulatory function of a coiled coil formed in PspA(1–144) [12,13], and (iii) the involvement of the N-terminal amphipathic helix AH1 in membrane surface interaction [16,12]. While a strong recombinant overproduction of full-length PspA leads to membrane localization of the recombinant protein and induction of the Psp system *in vivo* [17], PspA variants that lack the C-terminal region do not associate with membranes, nor do they induce the Psp response [13]. It was therefore proposed that membrane recruitment and oligomerization of PspA are linked processes that are required for the upregulation in response to membrane stress [16]. Based on these aspects, PspA has been dissected into distinct functional domains, with PspA(1–144) as the PspF interacting and regulating domain, and PspA(145–222) as the domain required for oligomerization and membrane interaction [15]. So far only negative evidence implied a role of the C terminal domain PspA(145–222) in oligomerization and membrane association [13], and its interactions have not been demonstrated. In this study, we analyzed the effects of PspA domains on the regulation of the key Psp response promoter, P_*pspA*_, we determined the localization of the respective domains under non-stress and stress conditions, and addressed the interactions of the domains with other PspA-domains as well as with PspF and with a Psp response inducing protein. The data indicate a novel function of C-terminal PspA fragment PspA(145–222) in PspF regulation, which relies on PspA/PspA interactions and PspA/PspF interactions that are mediated by this domain.

## Materials and methods

### Strains and growth conditions

*E*. *coli* strain MC3 [18] and its derivatives were used for all fractionation and co-elution experiments, as well as for P_*pspA*_ promoter activity quantifications. *E*. *coli* strain XL1-Blue Mrf’ Tet (Stratagene) was used for cloning. Cells were grown aerobically in LB medium (1% tryptone, 0.5% yeast extract, 0.5% NaCl) at 37°C with the appropriate antibiotics (100 μg/ml ampicillin, 25 μg/ml chloramphenicol, 50 μg/ml kanamycin). 0.5 mM IPTG or 0.1% rhamnose were used to induce P_*lacZ*_- or P_*rhaB*_-dependent protein production at indicated time points. *E*. *coli* strain BTH101 (Euromedex, Souffelweyersheim, France) was used for bacterial-2-hybrid studies and 0.1% (w/v) maltose-containing MacConkey agar plates supplemented with ampicillin and kanamycin were used for screening. Cells were grown for 72 h at 30°C.

### Construction of *psp* deletion strains

The Keio-collection strains BW25113 *pspA*::*kan* and BW25113 *pspF*::*kan* [19] were used to construct the strains MC3 *pspA*::*kan* and MC3 *pspF*::*kan* by phage transduction using P1_*vir*_ according to standard protocols [20]. After the initial selection on LB agar plates containing kanamycin (50 μg/ml), recipient clones were purified and the position of the kanamycin cassette was confirmed via colony PCR. The kanamycin cassette was removed using not nessearilypCP20-endcoded flippase according to the standard protocol of Datsenko and Wanner [21]. The loss of the kanamycin cassette was confirmed via colony PCR. For construction of the *E*. *coli* strain MC3 *pspFABCDE*::*kan* strain BW25113 *pspFABCDE*::*kan* was first constructed using the parental strain of the Keio-collection BW25113 [19] and the λ red recombinase system described by Datsenko and Wanner [21] using pKD4 as template and *pspF*-P1-F (CAC GCC GCA TCC GGC AAG TTG TAT TGC TCA ACT TCG GTG TAG GCT GGA GCT GCT TC) and *pspE*-P2-R (AAA ACG GCG CAT AAG CGC CGC TCA TGG TGA ATT CTT ATG GGA ATT AGC CAT GGT CC) as primer for amplification of the kanamycin cassette with flanking homologous regions that correspond to the flanking region of *pspF* and *pspE*. After transformation kanamycin-resistant clones were checked for loss of the pKD46 helper plasmid and positon of the kanamycin cassette were confirmed by colony PCR/sequencing. BW25113 *pspFABCDE*::*kan* were then used as donor for phage transduction of MC3 and the resulting kanamycin-resistant strain was checked again via PCR/sequencing.

### Plasmids

The construction of pBW-*tatA*-strep and pBW-*tatA*-NT-*mhip*-strep that are derivatives of the rhamnose inducible vector pBW22 [22], has been described previously [23]. To achieve a constitutive production of the C-terminal domain of PspA, we constructed the plasmid pABS-Ptat-H10*mhip*-*pspA*(CT) which encodes an N-terminal fusion of the C-terminal region of PspA to the mature domain of HiPIP, a tightly folded 9.6 kDa protein that we used to stabilize protein fragments [23]. An N-terminal decahistidine-tag (H10) was added via amplification of *mhip* with the primers NdeI-H10-*mhip*-F (ATA TAT CAT ATG CAT CAT CAC CAC CAC CAC CAC CAC CAC CAC TCC GCT CCC GCC AAT G) and *hip*-BamHI-R (ATA TAT AGG ATC CGC CGG CCT TCA GGG TC), using the plasmid pEXH5-tac as template [24]. The PCR product was cloned in the NdeI/BamHI sites of pABS-P*tat*-*pspC*-H6 [23], resulting in pABS-P*tat*-H10*mhip*-H6. The *pspA* C-terminal region was amplified using the primers *pspA145*-BglII-F (ATA TAT AGA TCT GCA AAC TCG TCG CGC G) and *pspA*-BamHI-R (ATA TAT GGA TCC TTA TTG ATT GTC TTG CTT C). The PCR product was restricted with BglII/BamHI and cloned into the BamHI site of pABS-P*tat*-H10*mhip*-H6. pABS-P*tat*-*pspA*-H6 and pABS-P*tat*-*pspA*(1–144)-H6 were constructed using NdeI-*pspA*-F (ACA ACC ATA TGG GTA TTT TTT CTC GCT TTG C) as forward primer and *pspA*-BamHI-R or *pspA*(144)-BamHI as reverse primer, respectively, and cloned in the NdeI/BamHI sites of the above described pABS-P*tat*-*pspC*-H6. All constructs were confirmed by sequencing. Plasmids and primers used for the bacterial-2-hybrid screen are listed in S1 Table. For a fast cloning procedure, plasmids pUT18C-*pspA*(25–144)-strep and pKT25-*pspA*(25–144)-strep were constructed first, using primers XbaIgBAmHI-*pspA*(25)-F (TAT AAT CTA GAG GGA TCC CCA CAG AAA CTG GTT CG) and *pspA*(144)ggKpnIstrepTAAEcoRI-R (TTA ATG AAT TCT TAT TTT TCG AAC TGC GGG TGG CTC CAG GGT ACC CCC TGA TGA CGT AAC ATC AA TG) to amplify *pspA*(25–144) encoding PspA(25–144) with a C-terminal *Strep*-Tag. XbaI and EcoRI were used for cloning of the PCR products into pKT25 and pUT18C. Plasmids pKN-strep-T25 and pU-strep-T18 were designed by amplifying pKNT25 and pUT18 with primers NdeI-strep-BamHI-pKTN25/pUT18-F (TTA ATC ATA TGT GGA GCC ACC CGC AGT TCG AAA AAG GAT CCA CCA TGA TTA CGC CAA G) and pKNT/pUT-NdeI-R (ATA TAC ATA TGT GTT TCC TGT GTG AAA TTG TTA TC). PCR products were cleaved with NdeI and religated. By using these plasmids, all fragments could be cloned and interchanged using the KpnI/BamHI sites.

For complementation of the minimal signaling cascade comprising PspF, PspA, PspB, and PspC, the *pspFpspABC* region was amplified using the primers PstI-strep-*pspF*-F (ATT ATC TGC AGC TAT TTT TCG AAC TGC GGG TGG CTC CAA ATC TGG TGC TTT TTC AAC) and *pspC*-BamHI-H6-PvuI-R (ATA TAT CGA TCG CTA GTG GTG GTG GTG GTG GTG GGA TCC CAG TTG ACG GAA ACG GC). The PCR product was cleaved with PstI/PvuI and ligated in the PstI/PvuI cleaved backbone of pUL-P*tat* [13] resulting in pUL-*pspF*strep-*pspABC*-H6. To construct pUL-*pspF*strep-*pspA*(1–144)*BC*-H6 the stop codon TAA was introduced at position PspA(A145) and the W56A mutation of PspF as well as the V105D mutation of PspC were introduced via QuikChange (Stratagene), using the primers listed in S2 Table. All constructs were confirmed by sequencing.

### Cell fractionation

For cell fractionation experiments, cells were aerobically grown at 37 °C in the presence of the appropriate antibiotics. If protein production was rhamnose-dependent, rhamnose was added to a final concentration of 0.1% (v/v) and cultivation proceeded for 3 h. IPTG was added to a final concentration of 0.5 mM for detection of T25 fusion proteins in the middle of the exponential phase, and cultivation was continued for 2 h. Cells were harvested via centrifugation at 4.500 x *g* for 10 min at 4°C. Cell densities of corresponding cultures were normalized prior to the centrifugation step. Cell pellets were suspended in 50 mM Tris HCl pH 8.0, 250 mM NaCl, and after adding DNaseI and 1 mM PMSF, cells were homogenized by ultrasonication. Cell debris was removed by centrifugation at 14.000 x *g* for 10 min at 4°C, and membrane and soluble fractions were prepared from the supernatant by ultracentrifugation at 130,000 x *g* for 30 min at 4°C. Fractions were analyzed by SDS-PAGE and Western blotting as described elsewhere.

### Co-elution assays

To analyze interactions between Strep-tagged TatA and PspA variants, co-elution experiments were carried out as described previously [23], with the exception that cell debris was removed prior to TatA-strep purification. For co-elution assays with His-tagged proteins, cells were resuspended in 100 mM Tris-HCl pH 8.0, 150 mM NaCl, 20 mM Imidazol, and disrupted via French Press, and affinity chromatography was carried out using the Protino Ni-NTA resin (Macherey-Nagel, Düren, Germany) with the above buffer system. After loading and 6 washing steps (each 1 column volume), His-tagged proteins were eluted by increasing the imidazole concentration to 250 mM. Elution fractions were analyzed via SDS-Page and Western blotting.

### β-Galactosidase assays

For the determination of the *pspA* promoter activity, LacZ assays were performed as described previously with strain MC3 and derivatives thereof [13]. All assays were done in triplicates. Some variability was observed with respect to absolute promoter activity levels between assays that were not carried out in parallel, and all data and error bars that are combined in diagrams are therefore from parallel assays. For induction of rhamnose- of IPTG-dependent expression systems, 0.1% rhamnose or 0.5 mM IPTG were added directly after inoculation, respectively. For determination of the reconstitution of the adenylate cyclase activity in BTH101, three independent clones were picked from the MacConkey agar plates and cultivated overnight in LB medium supplemented with 0.5 mM IPTG. LacZ activities of the overnight cultures were determined as described above.

## Results

### PspA(1–144) and PspA(145–222) can independently inhibit the AAA+ family enhancer binding protein PspF in the absence of full-length PspA

**Fig 1:**
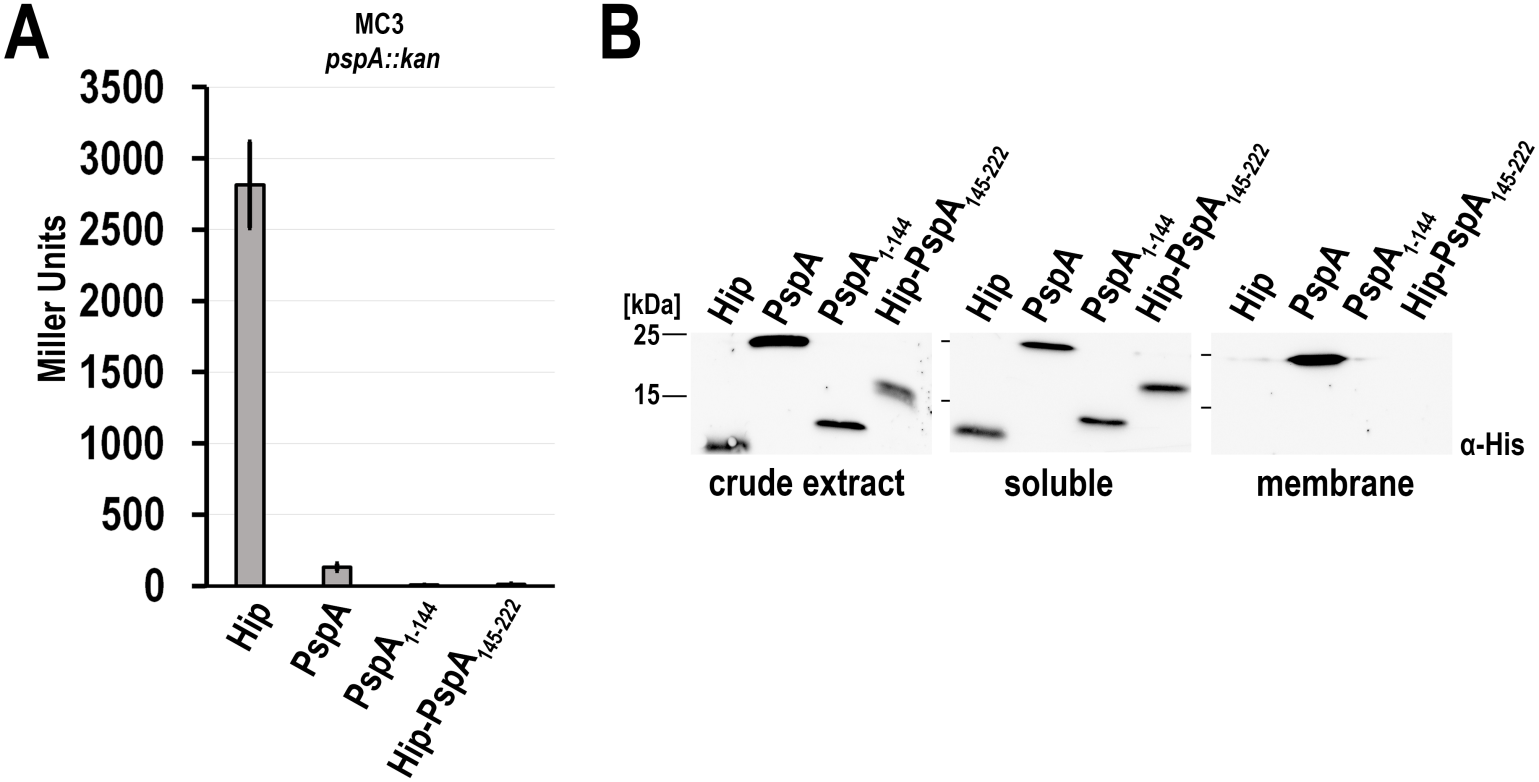
PspA(1–144) as well as Hip-PspA(145–222) can independently down-regulate PspF-dependent promoter activity. (A) LacZ activity assays with a *ΔpspA* reporter strain (MC3 *pspA*::*kan)*, in which the PspF regulated *pspA* promoter is fused the reporter gene *lacZ*. The strain was transformed with vectors for constitutive production of either HiPIP, PspA, PspA(1–144), or Hip-PspA(145–222). (B) Detection of the indicated hexahistdine-tagged proteins in crude extract and the soluble or membrane fractions of the strains used in (A) by SDS-PAGE/Western blotting. The optical density of all cultures was normalized pior to cell harvest. Blots were developed using specific His-Tag antibodies and ECL reaction.

PspA is known to have dual functions in distinct oligomerization states. It can multimerize and interact with membranes, and six PspA protomers can interact with hexameric PspF to regulate this σ^54^ activating enhancer binding protein [15]. The N-terminal domain of PspA, PspA(1–144), folds tightly to a coiled coil that is able to interact with PspF [13]. The N-terminal 22 residues of this domain fold back to the coiled-coil surface, and upon membrane surface interaction this region forms an amphipathic helix that is proposed to sense membrane stored curvature elastic stress [14,12]. The C-terminal domain, PspA(145–222), is required for oligomerization and thus likely mediates PspA-PspA interactions [15]. So far, no direct regulatory function has been attributed to the C-terminal domain. To assess the potential role of PspA(145–222) within the Psp regulatory cascade, we recombinantly produced this domain in a reporter strain that monitors the P_*pspA*_ promoter activity by a promoter *lacZ* reporter fusion. For comparison, we included also full-length PspA and PspA(1–144) that both are known to regulate PspF (Fig 1). To stabilize PspA(145–222) to a detectable level, we fused it to the C-terminus of HiPIP, an unrelated small globular protein that is used to stabilize fused protein fragments [23,25]. The resulting fusion protein, termed Hip-PspA(145–222), was stable and completely soluble (Fig 1B). As negative control, we also analyzed HiPIP alone in these experiments. All proteins were produced in comparable amounts (Fig 1B). As expected, recombinant full-length PspA could be detected in the soluble and the membrane fractions, whereas PspA(1-144) as well as HiPIP were only detectable in the soluble fraction (Fig 1B). We first carried out *pspA* promoter activity assays in a P_*pspA*_ *lacZ* reporter strain that had the chromosomal *pspA* gene deleted to avoid an interference of wt-PspA (Fig 1A). In the negative control, the strain that produced only HiPIP showed no reduction in LacZ activities, confirming that HiPIP has no influence on the regulatory cascade. In contrast, *pspA* promoter activity was almost completely downregulated by constitutive expression of full-length PspA or PspA(1–144). Full-length PspA suppressed PspF activity and thus complemented the PspA regulatory function in the mutant strain. With this expression system, PspA(1–144) silenced PspF to at least the extent of full-length PspA. To our surprise, we found a complete downregulation of PspF activity by Hip-PspA(145–222), which indicated that PspA(145–222) can silence PspF.

These findings suggested that not only the PspA(1–144) domain but also the PspA(145–222) domain can interact with PspF, and both domains contribute to its regulation.

### Several regions of PspA interact with PspF and silence its transcriptional activation

**Fig 2:**
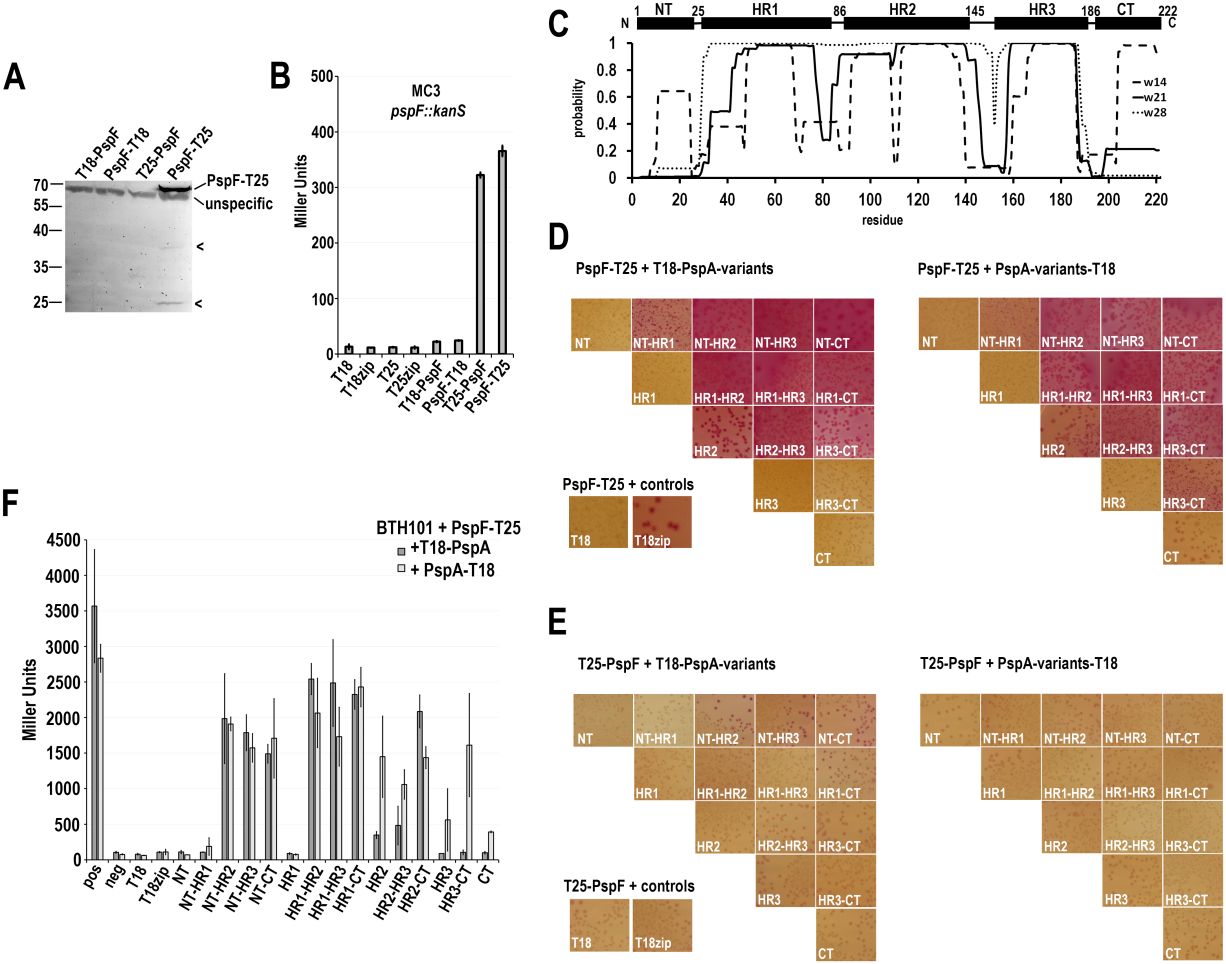
Not only the coiled coil domain of PspA(1–144) but also the C-terminal domain of PspA interacts with PspF. (A) SDS-PAGE/Western blot analysis of PspF fusion proteins used in the activity assay shown in (B). Western blots were developed using *Strep*-Tactin-AP conjugate (IBA) with subsequent alkaline phosphatase reaction for detection. The only detectable PspF fusion was PspF-T25. Other bands corresponded to an unspecific cross-reaction of the antibody (unspecific), and two degradation products (<). (B) LacZ activity assay of PspF fusion proteins in a reporter strain lacking PspF (*pspF*::*kanS*). Only T25 fusions of PspF were active and therefore used in the bacterial-2-hybrid screen. (C) Coils prediction of PspA using the COILS algorithm [26], and scheme including the PspA fragment start/end positions used in the B2H screen. (D/E) Bacterial-2-hybrid screen in *E*. *coli* strain BTH101 with PspF-T25 (D) or T25-PspF (E) in combination with the 15 indicated fragments of PspA fused N- or C-terminally to the T18 domain. Controls were performed using T18 and T18zip. (F) Quantification of LacZ activites of the B2H screen with indicated PspF-T25 and T18-PspA-variants. “pos”/”neg” indicate the positive (T18zip combined with T25zip) and negative controls as engineered by the manufacturer (T18 combined with T25, both without zipper fragment).

Knowing that PspA(145–222) is able to silence the Psp system in a *pspA* deletion strain, we performed a comprehensive bacterial-2-hybrid (B2H) screen to identify PspA fragments that interact with PspF. In this screen, inactive adenylate cyclase fragments (T18 and T25) can reconstitute adenylate cyclase activity when they are fused to peptides that interact. The cAMP production enables a fermentation of lactose in the medium. When grown on MacConkey agar, the acidification that results from lactose fermentation causes a deep red color of colonies and their surroundings and a precipitation of bile salts in the agar. If probed protein domains are not interacting, adenylate cyclase activity cannot be reconstituted and colonies do not metabolize the lactose of the medium, leading to small, beige colonies and a color shift to yellow that is caused by a basic pH. Based on the coiled-coil structure and further *in silico* predictions by the COILS algorithm of PspA secondary structure [26], we constructed 15 different PspA fragments that were C- or N-terminally fused to the T18 or T25 adenylate cyclase domains (Fig 2). Also PspF was fused C- or N-terminally as full-length protein to both protein domains. As only T25-fused PspF showed transcriptional activity in a Δ*pspF* reporter strain (Fig 2B), we used the functional T25-PspF and PspF-T25 constructs as bait in the B2H screen. We named the PspA fragments according to their known or predicted domain structure (Fig 2C): NT (N-terminal region) = Gly2—Pro25; HR1 (helical region 1, the first helix of the coiled coil that has been structurally solved) = Pro25—Glu85; HR2 (helical region 2, the second helix of the coiled coil that has been structurally solved) = Leu86—Gln144; HR3 (region including the predicted third helical region) = Ala-145—Ser-186; CT (C-terminal region) = Ser186—Gln222. The NT and CT regions may in principle also form helices with coiled coil tendency that are predicted by the COILS program when smaller window sizes are used (Fig 2C). The resulting names of the analyzed fragments are outlined in Table 1. We could only detect PspF/PspA fragment interactions with PspF-T25, which was also the only PspF fusion detectable by Western blotting (see Fig 2A). While the NT, HR1, HR3 and CT fragments did not exhibit any interaction with PspF-T25, the other fragments did interact (Fig 2D, 2E and 2F). The position of the T18 fusion (N- or C-terminal) was not relevant for the interactions of constructs that included the first two helical regions, i.e. the complete coiled coil domain of PspA. In contrast, the fusions to the single HR2 preferentially interacted as HR2-T18 fusion, and more clearly the HR3-T18 and especially the HR3-CT-T18 constructs interacted, whereas the N-terminal fusions showed no interaction (Fig 2D and 2F). All negative controls showed very low promoter activity (Fig 2F), but the T18zip construct resulted in reddish colonies, indicating a very high sensitivity of the growth phenotype on solid media. The interaction of the HR3-CT-T18 construct suggests that the observed regulation of PspF-dependent promoter activity (Fig 1) has been caused by a direct interaction.

**Table 1:** Abbreviations used for the bacterial-2-hybrid screen constructs.

Abbreviation	Start-End	Abbreviation	Start-End	Abbreviation	Start-End
**NT**	Gly2-Pro25	**HR1**	Pro25-Glu85	**HR2-HR3**	Leu86-Ser186
**NT-HR1**	Gly2-Glu85	**HR1-HR2**	Pro25-Gln144	**HR2-CT**	Leu86-Gln222
**NT-HR2**	Gly2-Gln144	**HR1-HR3**	Pro25-Ser186	**HR3**	Ala145-Ser186
**NT-HR3**	Gly2-Ser186	**HR1-CT**	Pro25-Gln222	**HR3-CT**	Ala145-Gln222
**NT-CT**	Gly2-Gln222	**HR2**	Leu86-Gln144	**CT**	Ser186-Gln222

**Fig 3:**
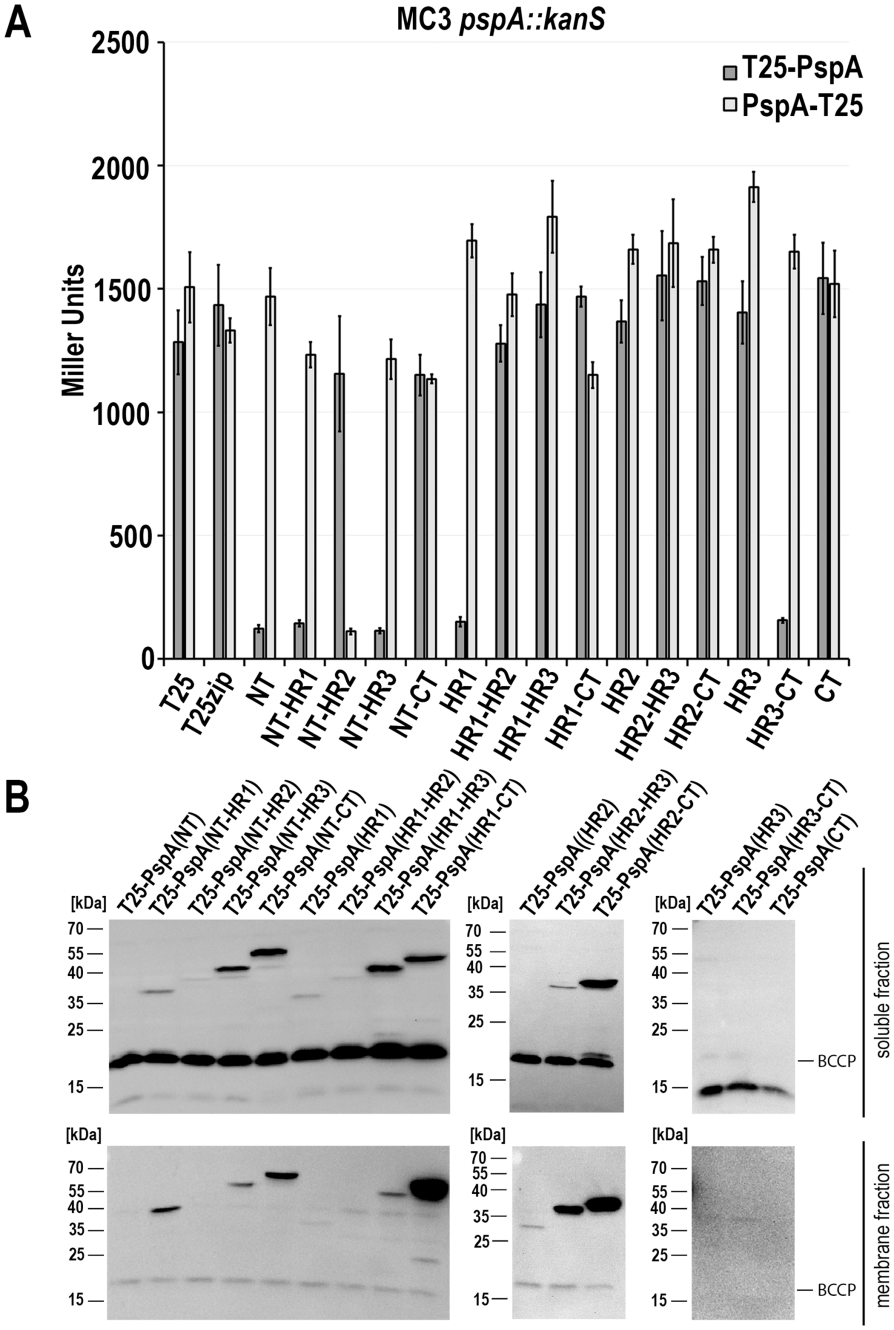
Not only N-terminal regions of PspA, but also Hip-PspA(145–222) inhibit PspF in a Δ*pspA* reporter strain. (A) LacZ activities of a *pspA*-deleted *pspA* promoter reporter strain (MC3 *pspA*::*kanS*) producing indicated PspA fragments N- (light grey) or C-terminally (dark grey) fused to the T25 domain. Protein production was induced by addition of 0.5 mM IPTG at the inoculation time. Inhibition of PspF activity could be observed for six fragments, namely T25-PspA(NT), T25-PspA(NT-HR1), PspA(NT-HR2)-T25, T25-PspA(NT-HR3), T25-PspA(HR1), and T25-PspA(HR3-CT). (B) Detection of T25-PspA-variants used in (A) in subcellular fractions by SDS-PAGE/Western-blotting. Among the N-terminal fusions to T25, only PspA(NT-CT)-T25 was detectable.

We then analyzed potential regulatory effects of all PspA fragments (Fig 3). As T18 fusions strongly affected growth of the promoter-*lacZ* reporter strain, we used the T25 fusions for these analyses. To ensure that an altered PspF activity only originated from an interaction of the recombinant proteins, we again used a *pspA* promoter activity reporter strain in which the chromosomal *pspA* gene was deleted. In this strain, high LacZ activities indicate that PspF activity is not affected by PspA fragments, whereas low LacZ activities indicate that PspA fragments inhibit PspF (Fig 3A). We could identify six PspA fragments that inhibited PspF activity (NT, NT-HR1, NT-HR2, NT-HR3, HR1, and HR3-CT). It was highly relevant whether the T25 domain was fused to the N- or the C-terminus of the respective fragment: The NT, NT-HR1, NT-HR3, HR1, and HR3-CT interacted only as N-terminal fusions, whereas the NT-HR2 construct interacted only as C-terminal fusion. Despite their strong interaction with PspF, fusions to full-length PspA (= the NT-CT construct) did not inhibit PspF activity, indicating that such fusions abolish the regulatory PspA function. We then examined whether the observed effects were due to a differential abundance of PspA fragments (Fig 3B). This was not the case, as protein levels did not correlate with inhibition of PspF activity. On one hand there were non-detectable constructs that showed a strong PspF inhibition and on the other hand fusions of T25 to full length PspA were highly abundant without having an inhibitory effect on PspF. It was recently shown by the Buck group that peptides of PspA_1-24_ and PspA_25-47_ inhibit AAA+ ATPase activity of PspF(1–275) [12]. In agreement with this study, the strong inhibition of the promoter activity by the PspA fragments containing the corresponding NT and HR1 regions now demonstrates that these fragments can indeed downregulate the *pspA* promotor *in vivo* (Fig 3). The inhibitory effect of T25-HR3-CT on PspF activity agrees with the same effect that had been already observed with the corresponding HiPIP fusion used for the initial analysis (Fig 1).

### PspA(1–144) and PspA(145–222) act differently in a PspA WT background

**Fig 4:**
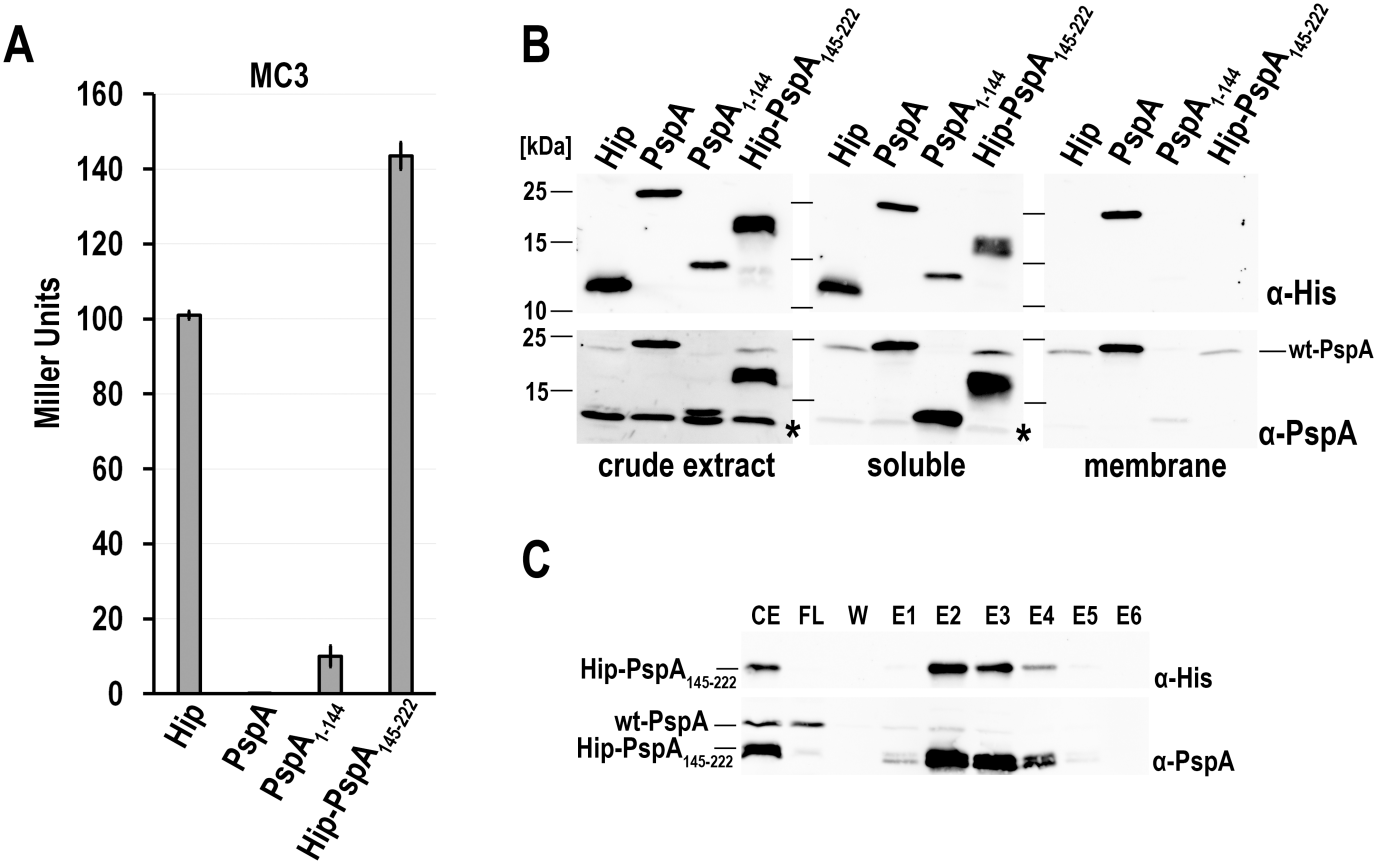
Hip-PspA(145–222) and PspA(1–144) have differential effects in a wild type *pspA* reporter strain. (A) LacZ activity assays of the *psp* wild-type reporter strain (MC3), constitutively producing either HiPIP, PspA, PspA(1–144), or Hip-PspA(145–222). (B) SDS-PAGE/Western blot detection of chromosomally encoded PspA (wt-PspA) and the recombinant PspA-variants used in the crude extract, soluble and membrane fractions of the strains used in (A). Blots were developed using specific antibodies recognizing the His-tag (upper panels) or PspA (lower panels, * indicates a cross-reaction of the PspA antibody). (C) Interaction of His-tagged Hip-PspA(145–222) with wt-PspA as examined by co-elution during affinity chromatography. CE = crude extract, FL = flow-through, W = last wash fraction, E1-E6 = elution fraction 1–6. Upper panel: detection of His-tagged Hip-PspA(145–222) by specific His-tag antibodies; Lower panel: detection by antibodies recognizing PspA.

After having established that PspA(145–222) and PspA(1–144) both inhibited PspF in a Δ*pspA* reporter strain, we analyzed the regulatory effects in a WT *pspA* background, i.e. in a reporter strain that is not mutated in *pspA* and thus contains the wild type *pspABCDE* operon (Fig 4A). We used the unrelated HiPIP as negative control and found that, as expected, HiPIP had no influence on the *pspA* promoter activity, as the basal activity level of the *pspA* promoter was not altered by HiPIP (~100 MU). Full-length PspA and PspA(1–144) resulted in silencing of the *pspA* promoter activity, indicating that these recombinantly produced proteins inhibited PspF very efficiently. Surprisingly, Hip-PspA(145–222) did not behave like full-length PspA or PspA(1-144) in this genetic background, and instead slightly induced the Psp response about 1.5-fold. As it could have been that the production of Hip-PspA(145–222) had triggered a degradation of full-length PspA, we examined the abundances of the respective recombinant constructs in subcellular fractions and assessed also the abundance of the non-recombinant wt-PspA (Fig 4B). As expected from previous studies [23], wt-PspA was detected in the soluble and in the membrane fraction in low amounts when the Psp system was not induced. Recombinantly produced His-tagged full-length PspA was also present in both fractions, whereas the well-characterized PspA(1–144) was almost completely soluble. In agreement with the promoter activity data (Fig 4A), both constructs repressed the production of wt-PspA to a hardly detectable level. Importantly, the production of Hip-PspA(145–222) did not trigger a membrane recruitment of wt-PspA in comparison to the negative control, nor did it cause a degradation of full-length PspA. Instead, we observed slightly higher levels of wt-PspA in the soluble fraction, indicating that the increased PspA levels were not targeted to the membrane under these conditions, i.e. in the absence of membrane stress. The upregulation could be explained by an interaction of His-tagged Hip-PspA(145–222) with wt-PspA, which was already indicated by the results of the two-hybrid assays (Fig 2), and we confirmed this interaction by affinity chromatography and co-elution analyses (Fig 4C). Although a large fraction of wt-PspA was not associated with Hip-PspA(145–222), some wt-PspA clearly co-eluted with Hip-PspA(145–222), indicating that PspA(145–222) can interact with full-length PspA, which supports the view that it may modulate the effect of full-length PspA on PspF activity.

### PspA(145–222) interacts with full-length PspA most likely by formation of antiparallel coiled coils

**Fig 5:**
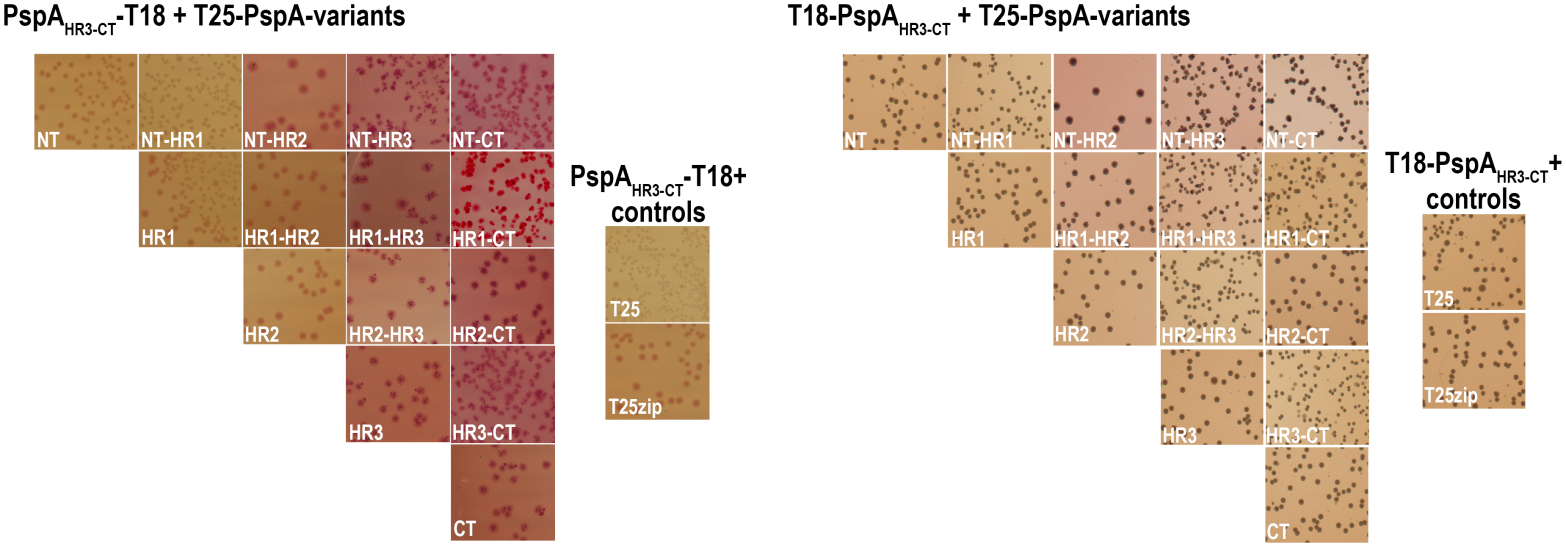
PspA(145–222) interacts most likely antiparallel with PspA domains. Bacterial-2-hybrid screen with HR3-CT-T18 (left side) or T18-HR3-CT (right side) and all 15 fragments of PspA as N-terminal T25 domain fusions in *E*. *coli* strain BTH101. Controls were performed using T25 and T25zip.

After having shown that PspA(145–222) can downregulate PspF activity as consequence of a direct interaction in the absence of full-length PspA (Figs 1–3), and after having shown that PspA(145–222) stimulates PspF activity in the presence of full-length PspA (Fig 4), we identified the region of PspA that interacts with PspA(145–222) by bacterial 2-hybrid analyses (Fig 5). We used the HR3-CT-T18 or T18-HR3-CT constructs as baits and systematically examined their interaction with all PspA fragments based on the above defined regions (NT/HR1/HR2/HR3/CT). While HR3-CT-T18 exhibited multiple interactions with full-length or truncated T25-PspA constructs (Fig 5, left panels), the T18-HR3-CT construct did not show strong interactions with any of the T25-PspA derivatives (Fig 5, right panels), indicating that an antiparallel setup (T18 fused to the C-terminus of PspA(145–222) as bait in combination with T25-fusions at the N-terminus of the prey) might be important for the interaction. N-terminal parts of PspA were not necessary for this interaction with PspA(145–222) (Fig 5, left panels). The adenylate cyclase activity could be reconstituted with HR3-CT-T18 in combination with T25 fusions to all fragments that included the HR3- or CT-regions. While the interaction with T25-HR3-CT was quite strong, the T25-HR3 or T25-CT constructs showed detectable but less interaction with HR3-CT-T18, suggesting that the HR3 and CT regions of PspA might interact together rather than individually with C-terminal regions of other PspA proteins. Little interaction was also obtained with the NT-HR2 fragment, which might add a level of complexity to the interactions if these weak interactions are physiologically relevant. However, the physiological function of the HR2/HR3-CT interaction has to be questioned, as the coiled coil of HR2 with HR1 is disrupted in that construct. In summary, the data support the view that PspA-PspA interactions are mediated via their C termini, and these interactions are likely antiparallel. As the C-terminal domain includes HR3, and as also the CT region might be able to form coiled coils, we suggest that this region possibly forms an antiparallel intermolecular coiled coil, which explains why the truncation of this domain results in soluble monomeric proteins [13,15].

### PspA(145–222) enhances a Psp response in a *psp* WT strain

**Fig 6:**
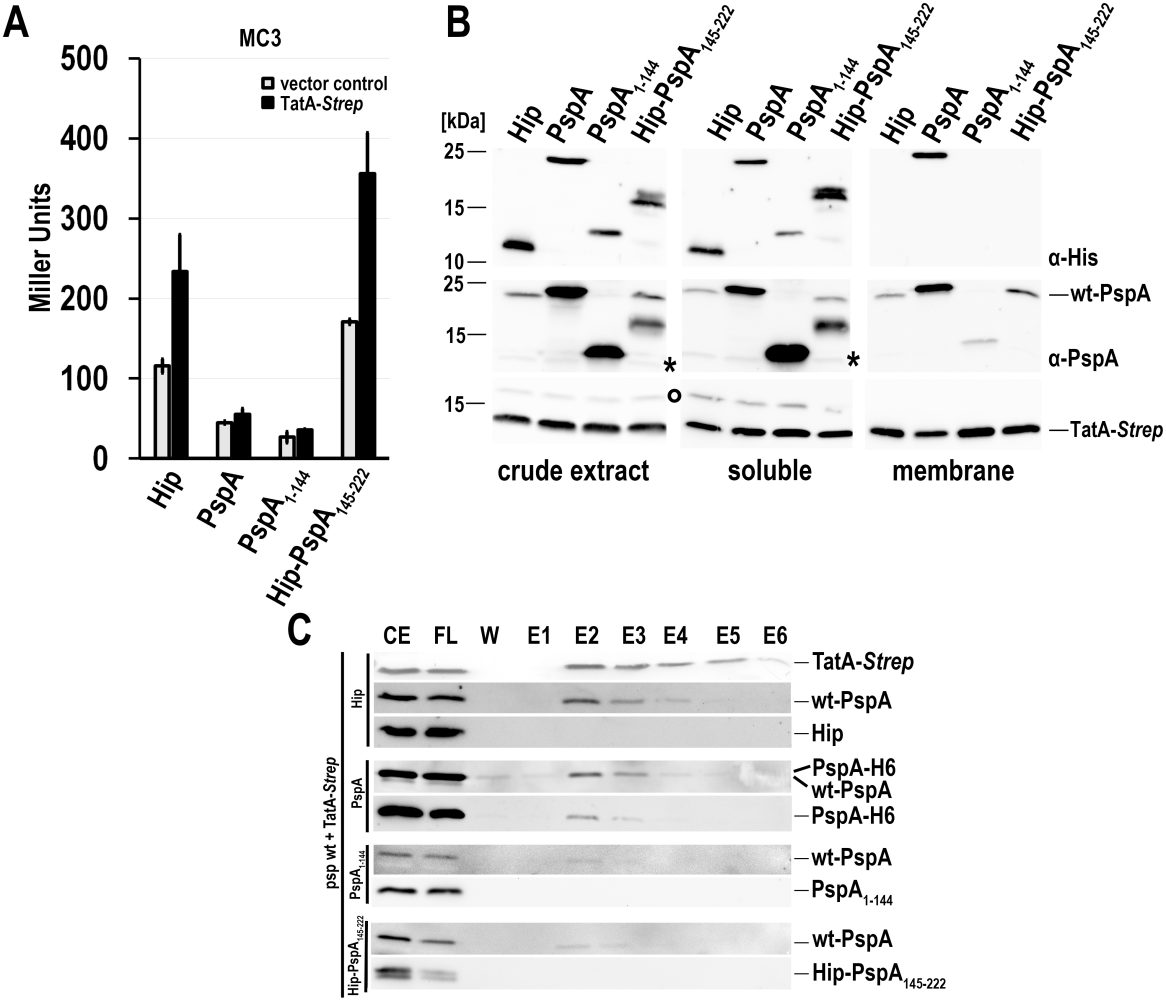
Inducibility of the Psp response in a *psp* wild type strain in the presence of additional recombinant PspA, PspA(1–144), or Hip-PspA(145–222). (A) LacZ activity assays of the psp wild-type reporter strain (MC3), constitutively producing either HiPIP, PspA, PspA(1–144), or Hip-PspA(145–222). Production of TatA-*Strep* was induced by 0.1% (v/v) rhamnose at the beginning of the cultivation. The strain containing the empty vector pBW22 was used as control. (B) SDS-PAGE/Western blot analysis of crude extract, soluble and membrane fractions of TatA-Strep producing strains used in (A), using for detection either specific His-antibodies (upper panels) or PspA antibodies (middle panels, * indicates a cross-reaction of the PspA antibody). TatA-Strep was detected using Strep-Tactin-HRP conjugate. “○”, biotin carboxyl carrier protein, BCCP (C) Interaction of His-tagged HiPIP, PspA, PspA(1–144), and Hip-PspA(145–222) with TatA-*Strep*, as evidenced by co-elution. TatA-Strep was purified via Strep-Tactin affinity chromatography. CE = crude extract, FL = flow-through, W = last wash fraction, E1-E6 = elution fraction 1–6. All fractions were analyzed by SDS-PAGE/Western blot using Strep-Tactin-AP conjugate for detection of TatA-Strep. Specific His-tag antibodies were used for detection of his-tagged HiPIP and PspA variants. Specific PspA antibodies were used for detection of wt-PspA and PspA variants. One representative detection of TatA-strep (Blot 1) is shown on the top. Blots 2, 4, 6, 9 (counted from the top) were developed with PspA antibodies, and Blots 3, 5, 7, 9 were developed using His-tag antibodies.

As PspA(145–222) stimulated the *pspA*-promoter activity in the presence of wt-PspA (Fig 4), we examined, whether membrane stress could induce the *pspA*-promoter activity on top of this stimulation. To induce a “complete” membrane stress Psp response involving all signaling components (i.e. PspA and the membrane components PspB and PspC), we produced the small membrane-anchored protein TatA [23]. TatA is part of the Tat translocase in *E*. *coli* and facilitates the translocation of folded proteins most likely by local destabilization of the lipid bilayer [25]. We thus produced TatA-*Strep* in the *pspA* promoter reporter strain in a *psp* wild type genetic background and in the presence of either PspA, PspA(1–144), or Hip-PspA(145–222), respectively. In a positive control, HiPIP was produced instead of any of the PspA constructs, which should not affect the Psp response induction by TatA. As expected, TatA induced the Psp response in the control strain (Fig 6A). In contrast, the promoter activity remained suppressed by recombinant PspA and PspA(1–144) when TatA-strep was produced, indicating that an increased abundance of these PspF-regulating proteins suppresses the Psp response. However, in the presence of Hip-PspA(145–222), which *per se* already increased the *pspA* promoter activity, TatA-*Strep* still induced a Psp response on top of this elevated level, indicating that the triggering of promoter activity by Hip-PspA(145–222) is unrelated to membrane stress and does not compromise the ability to respond to membrane stress.

When we examined the presence of the constructs in subcellular fractions, we found that wt-PspA could not be detected when full-length PspA or PspA(1–144) was recombinantly produced, confirming that the *pspA* promoter activity remains downregulated by highly abundant PspA or PspA(1–144) under membrane stress. This agrees with the data obtained in the absence of membrane stress (Fig 4) and with the promoter activity results (Fig 6A). Apparently, membrane stress sensing does not work when the PspF-inhibitory full-length PspA or PspA(1–144) are highly abundant.

It was shown in earlier studies that PspA interacts directly with the membrane protein TatA-*Strep*, which indicates a membrane interaction of PspA upon stress [23,25]. As TatA was *Strep*-tagged, we carried out co-elution experiments to address the question which fragments of PspA interact with TatA (Fig 6C). Notably, while full-length PspA co-eluted with TatA, neither the N- nor the C-terminal fragment of PspA could be detected in the elution fractions, possibly because these domains were not membrane-interacting (Fig 6B). The amount of full-length PspA that co-eluted with TatA-Strep appeared to be reduced in the presence of PspA(1–144) or Hip-PspA(145–222), but these domains obviously did not completely abolish the interaction of TatA-*Strep* with full-length PspA.

**Fig 7:**
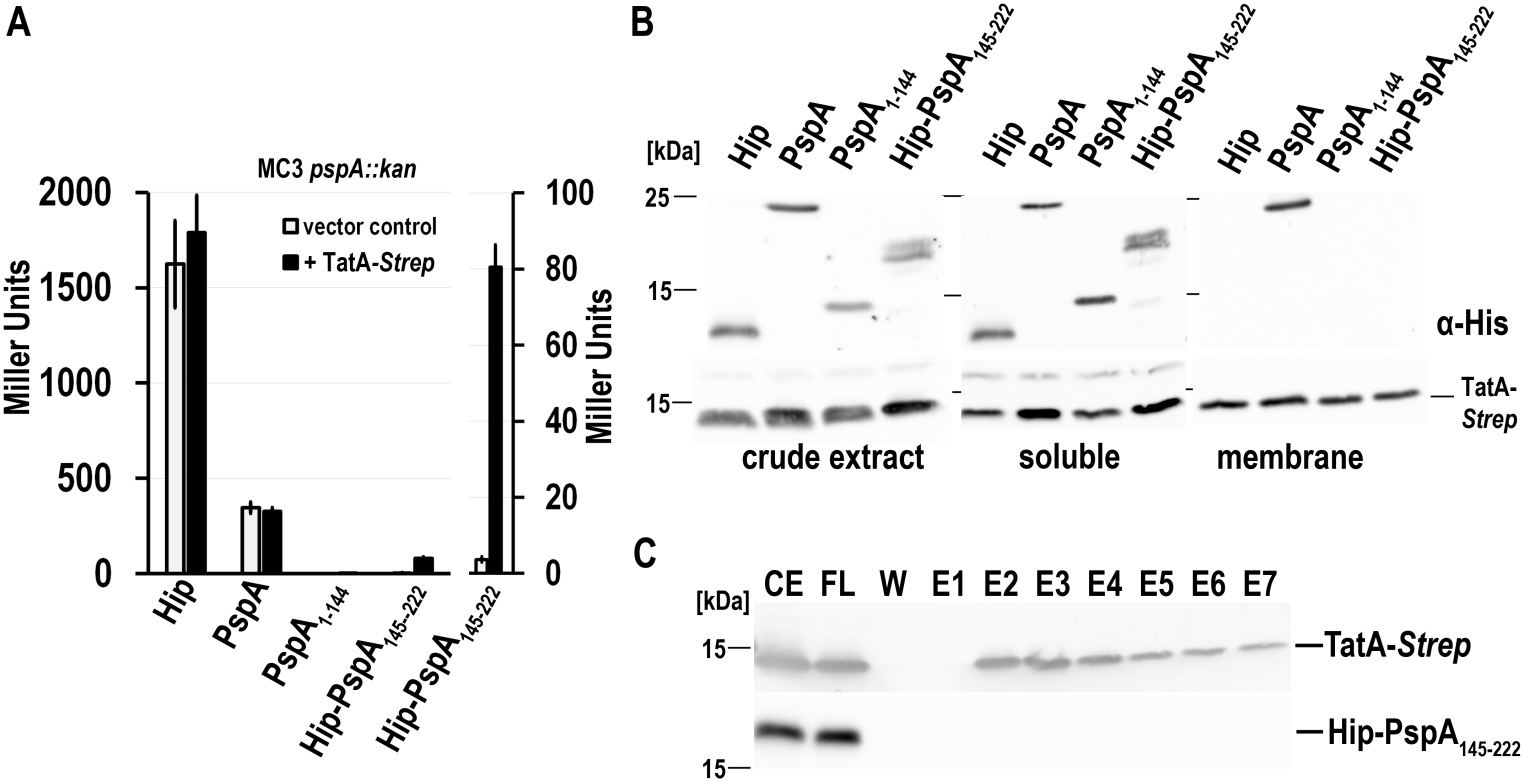
Examination of inducibility of the Psp response in a *pspA* deletion strain in the presence of additional recombinant PspA, PspA(1–144), or Hip-PspA(145–222). (A) LacZ activity assays of a *pspA* deletion reporter strain (MC3), constitutively producing either mature HiPIP, PspA, PspA(1–144), or Hip-PspA(145–222). Production of TatA-Strep was induced by 0.1% (v/v) rhamnose at the beginning of the cultivation. The strain containing the empty vector pBW22 was used as negative control. The graph on the right side shows a rescaled plot of the results next to it. (B) SDS-PAGE/Western blot analysis of crude extract, soluble and membrane fractions of TatA-*Strep* producing strains used in (A). Western blots were developed using His-tag (upper panels) or PspA (lower panels) specific antibodies, * indicates a cross-reaction of the PspA antibody). TatA-Strep was detected via Strep-Tactin-HRP conjugate. “○”, biotin carboxyl carrier protein, BCCP. (C) Coelution analysis of Hip-PspA(145–222) with TatA-*Strep* produced in the *pspA* deletion reporter strain as described in Fig 6.

We then did the same experiments in a *pspA* deletion background (Fig 7). The production of HiPIP, PspA or PspA(1–144) had the same effect on LacZ activity, no matter if TatA-*Strep* was present or not, as they silenced PspF transcriptional activity in both cases to the same extent. Again, PspA(1–144) was a stronger inhibitor than full-length PspA. In contrast, when TatA-*Strep* was produced in a strain that also produced Hip-PspA(145–222), the inhibitory effect of Hip-PspA(145–222) could be partially released, as evidenced by an increase of the *pspA* promoter activity from almost 3.5 Miller Units to an average of 80 Miller Units (Fig 7A). Compared to the negative control, the observed effect was not large in terms of absolute values, but it was significant and large in terms of relative increase. This finding was very interesting, as the effect could only be observed for Hip-PspA(145–222) and not for full-length PspA and PspA(1–144). Based on this, it may be speculated that PspA(145–222) is able to interact with TatA-*Strep* when wt-PspA is missing, but this interaction could so far not be demonstrated by co-elution analyses (Fig 7C), possibly indicating that PspA(145–222) is able to sense TatA induced membrane stress without the need of a direct interaction.

### PspF activity is PspC-independently upregulated in a system that lacks the C-terminal PspA domain

**Fig 8:**
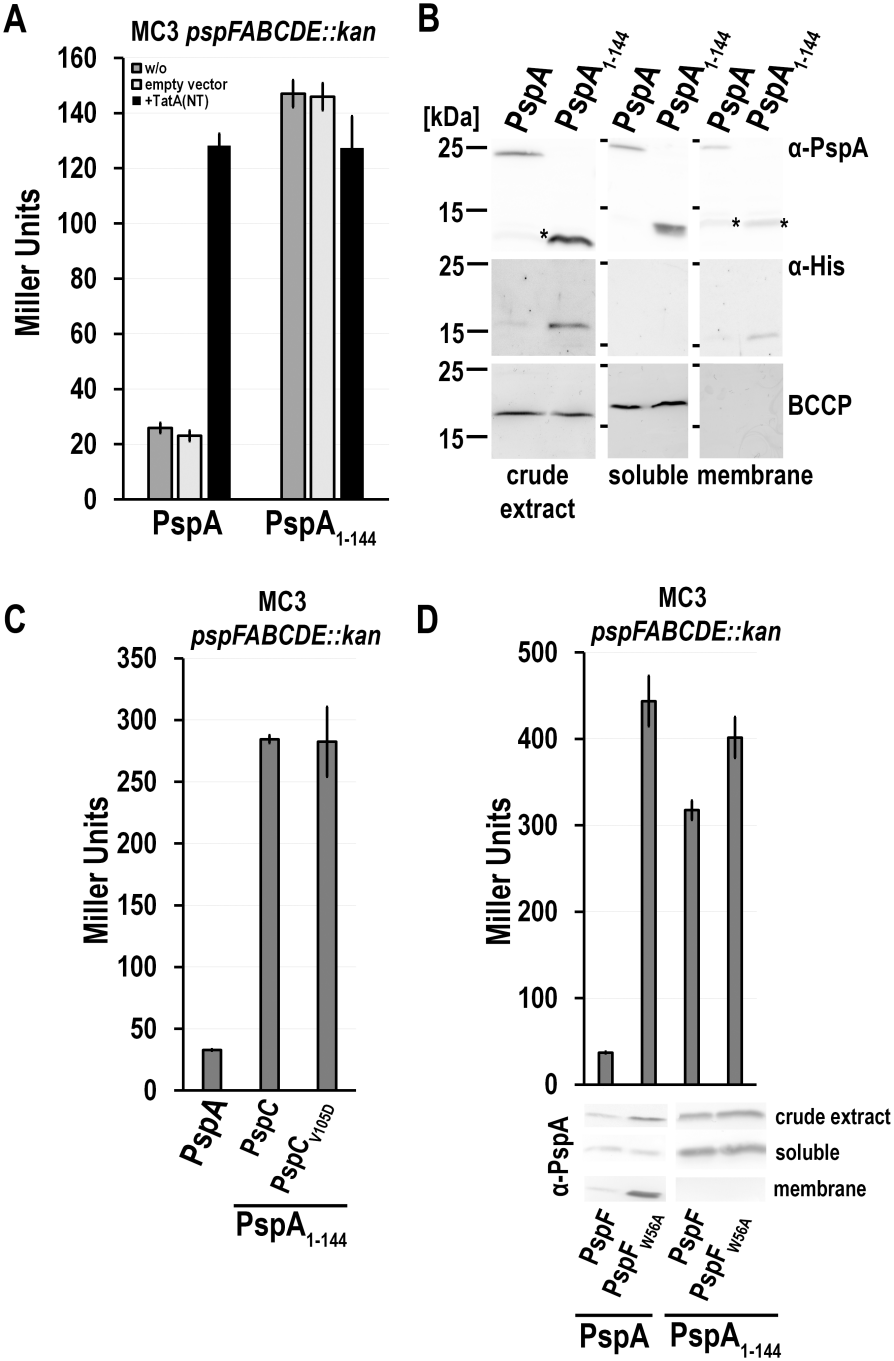
PspA(145–222) is necessary for keeping PspF activity at the basal level in a natively regulated Psp system. (A) LacZ activities of a *pspFABCDE* deletion P_*pspA*_ reporter strain MC3 *pspFABCDE*::*kan* with an in trans complemented minimal regulatory cascade using vector pUL-*pspF*strep-*pspABC*-H6 or pUL-*pspF*strep-*pspA*(1–144)BC-H6. Membrane stress was induced by rhamnose-dependent production of TatA(NT)-Hip. As negative controls, strains with the empty pBW22 vector and without the pBW22 vector (w/o) were used. (B) SDS-PAGE/Western blot analysis of crude extract, soluble and membrane fractions of the control strains (w/o) used in (A). PspA fragments were detected using PspA-specific antibodies (* = cross reaction of the PspA antibody). His-tagged PspC was detected via His-tag specific antibodies, and BCCP was detected as fractionation control with the Strep-tactin-AP conjugate. (C) LacZ activites of MC3 *pspFABCDE*::*kan* transformed with either pUL-*pspF*strep-*pspABC*-H6, pUL-*pspF*strep-*pspA*(1–144)BC-H6, or pUL-*pspF*strep-*pspA*(1–144)BC(V105D)-H6 for determination of PspC dependence of the results obtained in (B). (D) LacZ activites and detection of protein levels of MC3 *pspFABCDE*::*kan* transformed with either pUL-*pspF*strep-*pspABC*-H6, pUL-*pspF*(W56A)strep-*pspABC*-H6, pUL-*pspF*strep-*pspA*(1–144)BC-H6, pUL-*pspF*(W56A)strep-*pspA*(1–144)*BC*-H6. PspA detection in cellular fractions by SDS-PAGE/Western blot analysis, using PspA-specific antibodies.

We then addressed the question what happens in a system producing *pspFpspA*(1–144)*BC* in a natural regulatory cascade context. A *pspFpspABCDE* deletion mutant of the *pspA* promoter reporter strain MC3 was constructed and the unaltered *pspFpspABC* genomic region as well as the same region with a translational stop at codon 145 of PspA (= *pspFpspA*(1–144)*BC*) were introduced on an pSC101-based very low copy complementation vector [13] (Fig 8). The plasmid-encoded system had a lower basal activity than wild type but was fully functional, as PspF activity could be induced by production of the membrane anchor of TatA (Fig 8A), an established trigger of the Psp response [25]. However, despite PspA(1–144) was fully soluble and more abundant than full-length PspA, PspF activity was higher in the presence of PspA(1–144) than with full-length PspA (Fig 8B). It therefore appeared that the PspA(145–222) domain was needed for keeping PspF activity down in this system. Also the higher PspC levels in the strain producing PspA(1–144) reflect the increased promoter activity (Fig 8B). Importantly, the *pspFpspA*(1–144)*BC* system was not inducible by membrane stress (Fig 8A). In line with these observations, we found that the increased promoter activity in the PspA(1–144) system did not depend on a PspA/PspC interaction (Fig 8C), which is a key interaction for signaling of membrane stress [8,27]. As the Darwin group has shown recently that a V125C mutation of PspC from *Yersinia enterocolitica* abolishes the PspC/PspA interaction [8,27], we used the corresponding mutation in the *E*. *coli* system, PspC(V105D), to address the importance of the PspC/PspA interaction and found that it had no influence on PspF activity (Fig 8C). In the course of another project (manuscript in preparation), we had confirmed that, like in *Y*. *enterocolitica*, this mutation abolished PspC dependent signal transduction also in *E*. *coli*. A W56A mutation in PspF that is known to abolish PspA binding to PspF [5] resulted in an induction of the Psp response, and in case of the system with full-length PspA, PspA was recruited to the membranes (Fig 8D). As expected, in the system comprising PspA(1–144) instead of full-length PspA, the PspF(W56A) mutation compromized inhibitory functions of PspA(1–144) but did not result in any detectable membrane recruitment that appears to require the PspA(145–222) domain. Protein level corresponded to the observed promoter activities and hence it can be excluded that a lowered PspA or PspA(1–144) abundance caused the observed effects (Fig 8D). Together, these data indicate (i) that the PspA(145–222) domain in full-length PspA contributes to the inhibition of PspF by PspA at physiological abundances (Fig 8A and 8D), (ii) that PspA(1–144) alone cannot respond to known triggers of the Psp response (Fig 8A), (iii) that PspA(1–144) inhibits but does not silence PspF at physiological levels (Fig 8A), (iv) that the W56A mutation in PspF reduces the inhibition by full-length PspA and PspA(1–144), and (v) that the PspA(145–222) domain is required for membrane targeting (Fig 8D).

## Discussion

### The C-terminal domain of PspA can regulate PspF in a stress-responsive manner

The regulatory interactions of the Psp components PspA, PspB, PspC, and PspF in response to membrane stress are in the focus of most current research that is done in the Psp field. For *E*. *coli*, it was recently shown that six PspA(1–144) domains stably associate with hexameric PspF, exhibiting a slow dissociation kinetics [13], and that PspF can be regulated by PspA and PspA(1–144) *in vivo* and *in vitro* when it is bound to it [13]. It was therefore proposed that PspA may not need to dissociate from PspF to modulate its activity in response to stress signals. The ability to respond to membrane stress was not examined so far with the complex consisting of PspF and the C-terminally truncated PspA(1–144), and it was therefore unclear whether PspA(145–222) plays an active role in the regulatory cascade *in vivo*. However, it was known that the C-terminal domain of PspA is required for PspA oligomerization [15,5,13], and based on this evidence it has been postulated that it predominantly mediates oligomerization [15]. It was also clear from experiments with PspA(1–186) that the absence of the CT region abolishes the activation of PspF by PspA in the presence of membrane vesicles [14], and this domain was reported to be capable of inhibiting PspF ATPase activity [15]. However, the C-terminal domain is quite unstable *per se* and—albeit the data with truncated constructs suggested a role in regulation—positive data demonstrating a functionality directly were missing. We compared effects of recombinantely produced PspA(1–144), Hip-PspA(145–222), in which this domain is stabilized by an N-terminally fused protein, and full-length PspA on PspF regulation in an uninduced and induced state as well as in the presence or absence of native PspA. The stabilization by HiPIP or 2-hybrid domains was crucial for obtaining the *in vivo* data with PspA(145–222). Our observation of PspF inhibition by recombinant Hip-PspA(145–222) in the absence of full-length PspA now indicates a direct regulatory function of this domain *in vivo* (Figs 1, 4, 6, 7 and 8). The PspA(145–222) domain silences the *pspA* promoter in a mode that permits a significant activation by membrane stress as induced by TatA (Fig 7). This differs from the inhibitory effect of the recombinantly produced PspA(1–144) domain, as the PspA(1–144) regulated system does not respond to membrane stress anymore (Fig 7). Even the inhibition of PspF by recombinant full-length PspA was not affected when TatA-*Strep* was over-produced, indicating that the negative regulation was dominant under these conditions. Importantly, the PspA(1–144) construct includes an N-terminal amphipathic helix (residues 2–24) that is known to sense membrane stored curvature elastic stress [12], and therefore our data now indicate that this helix alone in conjunction with the regulatory coiled coil domain does not suffice for signaling of membrane stress to PspF. In agreement with this, it was reported that in a strain lacking genome-encoded PspA the Psp system is inducible by the pIV secretin when PspA(20–222), which lacks its N-terminal helix, is produced at low levels [16], indicating that this helix cannot constitute the only pathway for PspF activation. A crucial role of the distal C-terminus of PspA for the activation of the system was already postulated based on *in vitro* results that showed a requirement of the C-terminus for PspF activation [14]. As that study used truncated PspA, it remained unclear whether the absence of the C-terminus disabled stress sensing, or whether its lack only caused a dominant inhibition by the N-terminal PspA regions that are known to inhibit PspF [15,12,13,16]. The C-terminal region that starts behind the structurally solved N-terminal coiled coil, i.e. position 145, has not been analyzed so far and C-terminal fragments included regions of this N-terminal coiled coil, which are known to have effects on their own and thus compromise the interpretability of the data. Earlier *in vitro* and *in vivo* studies convincingly showed the contribution of the C terminal domain to oligomerization of PspA, but a direct influence on PspF activity by C terminal domains of PspA could not be determined as the used PspA fragments did neither inhibit nor interact with PspF *in vivo* and *in vitro* [15,5]. Our data are therefore first direct evidence for the involvement of C terminal domain of PspA in regulation and stress-sensing, and they support the proposed role of this domain [14].

It is intriguing that neither PspA(1–144) nor PspA(145–222) interact with TatA-*Strep*, albeit a Psp system down-regulated by PspA(145–222) is able to respond to the presence of this Psp response-inducing membrane protein. TatA-*Strep* causes this effect on PspF activity although it does not detectably interact with the PspA domains, which are not membrane associated. PspA(145–222) must be bound to the PspF hexamer to keep its activity at the observed very low level, and the activation suggests that there are transient interactions with the membrane that are below any detection limit, as the signal must be somehow transferred to PspFA. Such transient membrane interactions have been already postulated for the native PspFA complex [28,16]. It may be hypothesized that the TatA-generated stress signal induces interactions between PspA C-termini or other Psp system components that are responsible for the observed reduction of PspF inhibition. The nature of the membrane stress signal generated by TatA is so far unknown. On one hand, TatA is known to destabilize membranes, on the other hand it has been shown to interact with the Psp system [25,23]. It could be that TatA clusters can induce SCE stress, and this might be sensed by the system without direct TatA interactions [14]. As full-length PspA interacts with TatA, it is likely that this interaction can also contribute to sensing [23].

### Intermolecular interactions as mediated by PspA(145–222) may explain the enhancement of PspF activity in the presence of full-length PspA

Despite a clear downregulation of PspF by PspA(1–144) and PspA(145–222) in a Δ*pspA* background, the responsiveness of the PspA(145–222) inhibited system to the membrane stressor TatA already suggested that the interactions of PspA(1–144) and PspA(145–222) with PspF have distinct functions. Strikingly, PspA(145–222) weakly induced the promoter activity in the presence of wt-PspA, whereas PspA(1–144) still silenced the promoter under these experimental conditions (Figs 1 and 4). As the C-terminus is known to mediate oligomerization of PspA, we expected that the observed upregulation was due to an induction of wt-PspA oligomerization and a concomitant membrane localization. However, although more PspA was present in the cells as consequence of the upregulation, a shift to a membrane associated state did not occur and there was rather more soluble PspA (Fig 4). The increased levels of soluble PspA in response to PspA(145–222) did not lower the transcriptional activity of PspF. This contrasts the effect of increased levels of recombinant PspA or PspA(1–144), which result in a down-regulation of the *pspA* promoter activity (Fig 4). As PspA(145–222) interacts with itself and thus also with full-length PspA (Figs 4 and 5), we propose that the interaction of PspA(145–222) with PspF-bound wt-PspA affects the interaction of this PspA domain in wt-PspA with PspF, resulting in conformational changes of PspF-bound PspA and an increased PspF activity. We do not think that PspA(145–222) can induce a dissociation of wt-PspA from PspF, as this would consequently allow excess PspA(145–222) to associate with the liberated PspF, which would inhibit its activity, considering that PspA(145–222) silences PspF in the absence of PspA (Figs 1 and 3). PspA(145–222) might interact via formations of antiparallel coiled coils, as evidenced by the two-hybrid data (Fig 5), but this is certainly speculative, and the helical structure of this PspA domain remains to be experimentally confirmed.

### PspA(145–222) can mediate oligomerizations that may contribute to membrane interactions and Psp system inducibility

We noted that PspA(145–222) is completely soluble, although it can mediate PspA/PspA interactions (Figs 4 and 5). One explanation for this observation could be, that PspA can only interact with membranes when it contains its N-terminal region that can form an amphipathic helix [12]. Importantly, the monomeric PspA(1–144) is also soluble, indicating that single amphipathic helices are not stably membrane interacting *per se*. Under non-stress conditions, the monomeric PspA(1–144) proteins have their N-terminus folded back onto the surface of the coiled coil, which is proposed to be the “parking position” of that sensor region [13]. *In vitro*, PspA(2–24) turned into a helical structure in the presence of negatively charged phospholipids and it was able to inhibit the ATPase activity of PspF [12]. The same was reported for PspA(25–47). This stretch of residues includes residues whose PspF-regulatory function has been established [13]. Although it must be noted that in these studies this fragment was taken out of its stable coiled coil environment, it appears that already the single helix of that coiled coil can regulate PspF with its known regulatory functionalities. However, only the N-terminal amphipathic helix is an obvious candidate for a region that is mediating membrane interaction. It is therefore likely that the oligomerization as mediated by the C-terminus of PspA promotes efficient membrane interactions of the N-termini that can sense stress, as multiple weak membrane interactions are additive in the oligomer, which strengthens the membrane interaction of oligomeric full-length PspA. Another contribution to this interaction will certainly be the reported binding of PspA to the C-terminus of PspC [8].

### PspA(145–222) is necessary to keep PspF activity on its basal level in a native regulatory system

The Psp system and especially its upregulation currently becomes more complex than thought initially. The hypothesis that PspA and more specifically the PspA(1–144) fragment dissociates from PspF was challenged by the dissociation constant of a PspA(1–144)/PspF(1–265) complex of ~1 μM and a half-life time of the complex of ~43 minutes [13]. It has been proposed that a fast regulatory response might be possible without a dissociation of PspA from PspF. We observed that, at physiological levels, full-length PspA is much more inhibitory to PspF that the soluble PspA(1–144) domain, suggesting that the PspA(145–222) domain contributes to PspF inhibition by PspA (Fig 8), which agrees with interaction and regulation data (Figs 1–5).

Induction of the system by the N-terminal membrane anchor of TatA leads to an activation of PspF in the same range as observed for the activation by PspA(1–144). This could be a coincidence, but the reason might be that induction of the system leads to a release of the C-terminal part of PspA from its binding site on PspF. The induced state of the PspAF complex might be mimicked by the PspA(1–144)/PspF complex lacking the C-terminus of PspA. In agreement with this view would be that PspA(145–222), which alone is able to bind and silence PspF activity (Fig 1), can (i) enhance the activity of PspF in the presence of full-length PspA (Fig 4), (ii) bind to other PspA(145–222) domains (Fig 5), and (iii) respond to membrane stress (Fig 7). The mechanism by which the PspA(145–222) domain inhibits PspF is not necessarily the same as the inhibitory mechanism of the PspA(1–144) domain, which affects the ATPase activity [13]. In a nutshell, the regulation by the PspA(145–222) domain agrees with all current models of PspF regulation [16,8,13]. Future studies will hopefully clarify the exact role of the different PspA domains for signaling and activation of PspF in response to membrane stress.

## Supporting information

S1 Table Plasmids and primers used in the bacterial-2-hybrid assay. (DOCX)

S2 Table Primers used for QuikChange of pUL-*pspF*strep-*pspABC*-H6. (DOCX)
