# Supplementary material for: Evidence for a second regulatory binding site on PspF that is occupied by the C-terminal domain of PspA
Source: PLoS One. 2018 Jun 15;13(6):e0198564. doi: 10.1371/journal.pone.0198564 (PMC6003685; doi:10.1371/journal.pone.0198564)
Supplement: S1 Table — (DOCX) [file pone.0198564.s001.docx]

# Supporting Information

Evidence for a second regulatory binding site on PspF that is occupied by the C-terminal domain of PspA

**Eyleen S. Heidrich and Thomas Brüser**

**S1 Table: Plasmids and primers used in the bacterial-2-hybrid assay.**

| Plasmid | Primers used for cloning | Reference |
| --- | --- | --- |
| pUT18 | - | Euromedex |
| pUT18C | - | Euromedex |
| pUT18C-zip | - | Euromedex |
| pKNT25 | - | Euromedex |
| pKT25 | - | Euromedex |
| pKT25-zip | - | Euromedex |
| pKN-strep-T25  pU-strep-T18 | **NdeI-strep-BamHI-pKTN25/pUT18-F:** tta atc ata tgT GGA GCC ACC CGC AGT TCG AAA AAg gat ccA CCA TGA TTA CGC CAA G   **pKNT/pUT-NdeI-R**: ATA TAC ATA TGT GTT TCC TGT GTG AAA TTG TTA TC | This work |
| pUT18C-*pspA*(25-222)-strep  pKT25-*pspA*(25-222)-strep | **XbaIgBAmHI-pspA(25)-F**: TAT aaT CTA Gag GGA TCC CCA CAG AAA CTG GTT CG  ***pspA*(144)ggKpnIstrepTAAEcoRI-R**: tta atG AAT TCT TAT TTT TCG AAC TGC GGG TGG CTC CAG GGT ACC ccC TGA TGA CGT AAC ATC AA TG | This work |
| pUT18C-*pspA*(2-25)-strep  pU-strep-*pspA*(2-25)-T18  pKT25-*pspA*(2-25)-strep  pKN-strep-*pspA*(2-25)-T25 | **BamHI-*pspA*(2)-F**: ttt aag gat ccg GTA TTT TTT CTC GCT TTG CCG  ***pspA*(25)ggKpnI:R**: TAT AAG GTA CCC CTG GAT CTT CCG CTT TCT CTA AC | This work |
| pUT18C-*pspA*(2-85)-strep  pU-strep-*pspA*(2-85)-T18  pKT25-*pspA*(2-85)-strep  pKN-strep-*pspA*(2-85)-T25 | **BamHI-*pspA*(2)-F:** ttt aag gat ccg GTA TTT TTT CTC GCT TTG CCG  ***pspA*(85)ggKpnI-R**: TAT AAG GTA CCC CAT CCT CTC TCT CTT TCA GC | This work |
| pU-strep-*pspA*(2-144)-T18  pKN-strep-*pspA*(2-144)-T25 | **BamHI-*pspA*(2)-F**: ttt aag gat ccg GTA TTT TTT CTC GCT TTG CCG  ***pspA*(144)ggKpnI-R:** TTA ATG GTA CCC CCT GAT GAC GTA ACA TCA ATG | This work |
| pUT18C-*pspA*(2-186)-strep  pU-strep-*pspA*(2-186)-T18  pKT25-*pspA*(2-186)-strep  pKN-strep-*pspA*(2-186)-T25 | **BamHI-*pspA*(2)-F:** ttt aag gat ccg GTA TTT TTT CTC GCT TTG CCG  ***pspA*(186)ggKpnI-R**: TTA TAG GTA CCC CGC TGT GGC TTT CTG CTT CCG C | This work |
| pUT18C-*pspA*(2-222)-strep  pU-strep-*pspA*(2-222)-T18  pKT25-*pspA*(2-222)-strep  pKN-strep-*pspA*(2-222)-T25 | **BamHI-*pspA*(2)-F**: ttt aag gat ccg GTA TTT TTT CTC GCT TTG CCG  ***pspA*(222)ggKpnI-R**: AAT TTG GTA CCC CTT GAT TGT CTT GCT TCA TTT TG | This work |
| pUT18C-*pspA*(25-85)-strep  pU-strep-*pspA*(25-85)-T18  pKT25-*pspA*(25-85)-strep  pKN-strep-*pspA*(25-85)-T25 | **BamHI-*pspA*(25)-F**: ttt aag gat ccC CAC AGA AAC TGG TTC G  ***pspA*(85)ggKpnI-R**: TAT AAG GTA CCC CAT CCT CTC TCT CTT TCA GC | This work |
| pUT18C-*pspA*(25-144)-strep  pU-strep-*pspA*(25-144)-T18  pKT25-*pspA*(25-144)-strep  pKN-strep-*pspA*(25-144)-T25 | **BamHI-*pspA*(25)-F:** ttt aag gat ccC CAC AGA AAC TGG TTC G  ***pspA*(144)ggKpnI-R**: TTA ATG GTA CCC CCT GAT GAC GTA ACA TCA ATG | This work |
| pUT18C-*pspA*(25-186)-strep  pU-strep-*pspA*(25-186)-T18  pKT25-*pspA*(25-186)-strep  pKN-strep-*pspA*(25-186)-T25 | **BamHI*-pspA*(25)-F:** ttt aag gat ccC CAC AGA AAC TGG TTC G  ***pspA*(186)ggKpnI-R:** TTA TAG GTA CCC CGC TGT GGC TTT CTG CTT CCG C | This work |
| pUT18C-*pspA*(25-222)-strep  pU-strep-*pspA*(25-222)-T18  pKT25-*pspA*(25-222)-strep  pKN-strep-*pspA*(25-222)-T25 | **BamHI-*pspA*(25)-F:** ttt aag gat ccC CAC AGA AAC TGG TTC G  ***pspA*(222)ggKpnI-R**: AAT TTG GTA CCC CTT GAT TGT CTT GCT TCA TTT TG | This work |
| pUT18C-*pspA*(86-144)-strep  pU-strep-*pspA*(86-144)-T18  pKT25-*pspA*(86-144)-strep  pKN-strep-*pspA*(86-144)-T25 | **BamHI-*pspA*(86)-F:** ttt aag gat ccC TGG CAC GTG CAG CGT TAA TTG  ***pspA*(144)ggKpnI-R**: TTA ATG GTA CCC CCT GAT GAC GTA ACA TCA ATG | This work |
| pUT18C-*pspA*(86-186)-strep  pU-strep-*pspA*(86-186)-T18  pKT25-*pspA*(86-186)-strep  pKN-strep-*pspA*(86-186)-T25 | **BamHI-*pspA*(86)-F:** ttt aag gat ccC TGG CAC GTG CAG CGT TAA TTG  ***pspA*(186)ggKpnI-R:** TTA TAG GTA CCC CGC TGT GGC TTT CTG CTT CCG C | This work |
| pUT18C-*pspA*(86-222)-strep  pU-strep-*pspA*(86-222)-T18  pKT25-*pspA*(86-222)-strep  pKN-strep-*pspA*(86-222)-T25 | **BamHI-*pspA*(86)-F**: ttt aag gat ccC TGG CAC GTG CAG CGT TAA TTG  ***pspA*(222)ggKpnI-R:** AAT TTG GTA CCC CTT GAT TGT CTT GCT TCA TTT TG | This work |
| pUT18C-*pspA*(145-186)-strep  pU-strep-*pspA*(145-186)-T18  pKT25-*pspA*(145-186)-strep  pKN-strep-*pspA*(145-186)-T25 | **BamHI-*pspA*(145)-F:** ttt aag gat ccG CGG CAA ACT CGT CGC GCG ATG  ***pspA*(186)ggKpnI-R**: TTA TAG GTA CCC CGC TGT GGC TTT CTG CTT CCG C | This work |
| pUT18C-*pspA*(145-222)-strep  pU-strep-*pspA*(145-222)-T18  pKT25-*pspA*(145-222)-strep  pKN-strep-*pspA*(145-222)-T25 | **BamHI-*pspA*(145)-F**: ttt aag gat ccG CGG CAA ACT CGT CGC GCG ATG  ***pspA*(222)ggKpnI-R**: AAT TTG GTA CCC CTT GAT TGT CTT GCT TCA TTT TG | This work |
| pUT18C-*pspA*(145-222)-strep  pU-strep-*pspA*(145-222)-T18  pKT25-*pspA*(145-222)-strep  pKN-strep-*pspA*(145-222)-T25 | **BamHI*-pspA*(145)-F**: ttt aag gat ccAGC TTC GGT AAA CAA AAA TCG  ***pspA*(222)ggKpnI-R**: AAT TTG GTA CCC CTT GAT TGT CTT GCT TCA TTT TG | This work |
| pUT18C-*pspA*(186-222)-strep  pU-strep-*pspA*(186-222)-T18  pKT25-*pspA*(186-222)-strep  pKN-strep-*pspA*(186-222)-T25 | **BamHI-*pspA*(186)-F:** tta aGG ATC CAG CTT CGG TAA ACA AAA ATC G  ***pspA*(222)ggKpnI-R**: aat ttG GTA CCc cTT GATT GTC TTG CTT CAT TTT G | This work |
| pUT18C-*pspF* strep  pU-strep-*pspF-*T18  pKT25-*pspF*-strep  pKN-strep-*pspF*-T25 | **BamHI-*pspF*(2)-F:** TTT AAG GAT CCG CAG AAT ACA AAG ATA ATT TAC  ***pspF*(325)ggKpnI-R:** TAT AAG GTA CCC CAA TCT GGT GCT TTT TCA ACA ACG C | This work |
