# Supplementary material for: Evidence for a second regulatory binding site on PspF that is occupied by the C-terminal domain of PspA
Source: PLoS One. 2018 Jun 15;13(6):e0198564. doi: 10.1371/journal.pone.0198564 (PMC6003685; doi:10.1371/journal.pone.0198564)
Supplement: S2 Table — (DOCX) [file pone.0198564.s002.docx]

# Supporting Information

Evidence for a second regulatory binding site on PspF that is occupied by the C-terminal domain of PspA

**Eyleen S. Heidrich and Thomas Brüser**

**S2 Table: Primers used for QuikChange of pUL-*pspF*strep-*pspABC*-H6**

| Exchange | Primers used for QuikChange | Reference |
| --- | --- | --- |
| PspF(W56A) | **PspF(W56A)-F**: CAT TAT CTC TCC TCC CGT GCG CAA GGG CCG TTT ATT TC **PspF(W56A)-R**: GAA ATA AAC GGC CCT TGC GCA CGG GAG GAG AGA TAA TG | This work |
| PspA(A145TAA) | **PspA(A145TAA)-F:** GCA TTG ATG TTA CGT CAT CAG TAA GCA AAC TCG TCG CGC GAT GTG  **PspA(A145TAA)-R:**CAC atc gcg cga cga gtt tgc tta ctg atg acg taa cat caa tgc | This work |
| PspC(V105D) | **PspC(V105D)-F**: CGA GAT GGA ACG TTA TGA TAC TTC CGA TAC TTT CAC G  **PspC(V105D)-R:** CGT GAA AGT ATC GGA AGT ATC ATA ACG TTC CAT CTC G | This work |
